# Insomnia in Postmenopausal Women: How to Approach and Treat It?

**DOI:** 10.3390/jcm13020428

**Published:** 2024-01-12

**Authors:** Gyun-Ho Jeon

**Affiliations:** Department of Obstetrics and Gynecology, Haeundae Paik Hospital, Inje University School of Medicine, Busan 48108, Republic of Korea; jeon285@hotmail.com; Tel.: +82-51-797-2020

**Keywords:** hot flushes, insomnia, menopause, sleep disorder, menopausal hormonal therapy

## Abstract

Insomnia is one of the major complaints of menopausal women with advancing age and may be complexly related to a variety of causes. However, there is still a lack of standards on the general approach and treatment for insomnia in menopausal women. The aim of this review is to summarize recent pathogenic theories of sleep disturbance in the menopausal period and discuss the approach and management of insomnia in postmenopausal women. Sleep disturbances in menopausal women may be associated with physical and psychiatric factors and other comorbid diseases. Careful history taking and multidisciplinary physical and psychosocial evaluation are necessary and, in particular, comorbidities related to sleep disorders, such as obstructive sleep apnea, must be taken into consideration. A unique aspect of insomnia in postmenopausal women is that menopausal symptoms due to hormonal decline can be closely related to sleep disturbances. Therefore, menopausal hormone therapy (MHT) should be considered as the treatment of choice among pharmacological treatments following cognitive behavioral therapy, which is suggested as the first-line treatment in the general population insomnia treatment guidelines. Additionally, melatonin and 5HT-based drugs, which have fewer side effects, along with MHT should be preferentially recommended in menopausal women.

## 1. Introduction

With the decline of reproductive hormones in the menopausal transition period, a substantial number of women experience physiological and psychological changes. Sleep disturbance is one of the major complaints of menopausal women with advancing age. Menopausal women also frequently experience other typical menopausal symptoms, including hot flashes (HFs), night sweats, palpitations, mood changes, anxiety, and depression, which also increase the risk of developing sleep problems [1]. Indeed, various sleep problems, such as decreased sleep duration, poor sleep quality, and early morning awakenings, commonly begin in the menopausal period [2]. It is reported that women in menopausal transition or menopause suffer from sleep disturbance or insomnia, ranging from 35% to 60%, and a significant number of women experience severe symptoms that impair daytime functioning [3]. Sleep disturbance can cause fatigue, somnolence, mood disorders, memory impairment, lack of attention, and even accidents, which can lead to behavioral, occupational, and social problems [4]. Recent studies revealed that insomnia is also associated with significant medical problems, such as cardiovascular disease, diabetes, and an increased risk of mortality [5]. In addition to menopause, it has been reported that women have specific periods related to vulnerability to sleep disorders, such as the menstrual cycle and pregnancy, suggesting a link between sleep disorders and female hormones [6,7]. As such, insomnia is closely related to hormonal changes and, although it is a major menopausal symptom, there are currently no universal guidelines for treating insomnia in menopausal women. Recently, Proserpio et al. also reviewed the mechanism and treatment of insomnia in menopausal women, with similar content to this review [8]; however, this review categorizes the causes and treatment of sleep disorders in menopausal women to make it easier to approach clinically and contains updated contents on menopausal hormone therapy (MHT), melatonin, and orexin antagonist. This review will summarize recent pathogenic theories of sleep disturbance, including hormonal changes during the menopausal period, and discuss the approach and management of insomnia in postmenopausal women.

## 2. Insomnia and Sleep Disorders: Definition

Insomnia is defined as difficulties falling asleep or maintaining sleep, which result in daytime impairment, despite adequate opportunity and circumstances to sleep. Chronic insomnia disorder is defined when it occurs at least three times per week for three months, according to the International Classification of Sleep Disorders, Third Edition (ICSD-3). Insomnia was traditionally approached as a primary or secondary (comorbid) disorder, provoked by physical problems or psychosocial factors, etc. (as will be discussed later in the Insomnia Etiology Section), but there were issues of uncertainty with the nature of the associations and the direction of causality in comorbid insomnia cases; thus, all insomnia diagnoses were consolidated under chronic insomnia disorder [9]. In addition to insomnia, obstructive sleep apnea (OSA), which is classified as a sleep-related breathing disorder, restless legs syndrome (RLS), and periodic movement disorder are major certain comorbid diseases encountered in sleep disorders in postmenopausal women. Indeed, insomnia and sleep-related breathing disorders are the two most common sleep disorders [9,10]. Other sleep disorders that are less common or unrelated in menopause include circadian rhythm sleep–wake disorder, narcolepsy, idiopathic hypersomnia, cataplexy, night terrors, parasomnias, and sleep paralysis [11].

## 3. Sleep Disturbance across Menopause: Epidemiology

Middle-aged women have increasing complaints of sleep disorders as they enter menopausal transition and the menopausal period. The incidence rates of sleep problems show 39–47% in peri-menopausal and 35–60% in postmenopausal women, compared to 16–42% in premenopausal women [3]. Although sleep deteriorates with age and is affected by many physical problems (lower back pain, musculoskeletal disorders, urinary symptoms, hot flushes (HFs), etc.), mood disorders and psychosocial factors, socioeconomic, and racial/ethnic factors [10,12], an independent relationship between menopausal stages and sleep disturbance, controlled for the effects of aging and other confounders, was shown in a meta-analysis of 24 cross-sectional studies [13]. In addition, the incidence of sleep-related breathing and movement disorders also increases in postmenopausal women due to age and obesity, as discussed later.

Meanwhile, women who had undergone surgical menopause and were not taking hormone therapy had the highest prevalence of sleep disturbance compared with natural menopausal transitional women, independent of age or years since surgery. The most common sleep complaint in these women was reported as frequent awakenings during sleep in the longitudinal analysis of the Study of Women’s Health Across the Nation (SWAN) [3]. Regarding the predictors of poor sleep quality during menopause, it has been reported that depressive symptoms, personal crisis, perceived health impairments, and frequent night sweats are related [14]. In a cohort study that followed a population-based sample for over one year, Lebland et al. reported that the greatest risk of developing insomnia was a previous insomnia episode, and thus premenopausal sleep status can also be considered as an important factor in predicting postmenopausal insomnia [15].

## 4. Insomnia and Menopause: Pathogenesis and Etiology

Sleep disturbance in postmenopausal women is pathophysiologically multifactorial (Table 1). Physiologically, it may be strongly associated with menopause symptoms, such as HFs and night sweats that can be experienced along with female hormonal changes [16], and psychiatrically, with mood disorders, anxiety, and depression [10]. In addition, family/economic/social stress, obesity, ill health, and drug and alcohol intake are common causes of sleep problems in middle-aged women, and commonly encountered comorbid diseases with sleep disorders include OSA, RLS, and periodic leg movement syndrome. After menopause, the prevalence of OSA increases due to weight gain and changes in fat distribution from increased testosterone production and decreased female hormones, and the incidence of RLS increases [14,17]. There is limited data showing that basic physiological changes, such as alterations in the circadian system and decreased melatonin secretion due to aging itself, also contribute to sleep difficulties in menopause [18].

### 4.1. Reproductive Hormonal Changes

Previous studies have reported beneficial effects of female sexual hormones on sleep. Estrogen blocks wake-promoting neurotransmitters, such as acetylcholine, histamine, norepinephrine, serotonin, and dopamine [19], and is known to have a thermoregulatory effect of regulating the lowest body temperature during the night, which provides good conditions for falling asleep [20]. Overall, estrogen seems to increase the rapid eye movement sleep and total sleep time and decrease sleep latency and awakenings after sleep [21]. Estrogen may also exert an antidepressant effect by regulating 5HT [22]. Progesterone stimulates benzodiazepine receptors, causing the release of gamma-aminobutyric acid (GABA), a sedating neurotransmitter, and thus induces sleep favoring non-rapid eye movement sleep [23,24]. Progesterone is also known to exert an anxiolytic and respiratory stimulant effect [24,25], which may also help promote good sleep.

Several population studies showed an association between reproductive hormone levels, including estradiol, FSH, and inhibin B, and sleep quality or sleep disruption in menopausal transition women [3,17,26,27,28,29]; however, their findings are inconsistent, and correlation with objective polysomnographic indices has also not been clearly demonstrated. The isolation of hormonal effects seems to be challenging because there are multiple influencing factors, such as high variability of their measurements across the menopausal transition.

### 4.2. Vasomotor Symptoms

HFs are a physiological result of peripheral and central temperature increases due to lowered estrogen levels. HFs are common complaints, reported by up to 80% of menopausal women [30], accompanied by increased body temperature during the nighttime and night sweats, which lead to sleep disturbance. A recent study found that 69.4% of HFs interfered with sleep [31], and an increase in HFs was common during early sleep (N1) and wake, typically preceding or occurring simultaneously with nighttime awakenings [32]. The SWAN data showed that women with moderate to severe HFs had a higher risk of frequent nocturnal awakenings compared to women without HFs [33]. Indeed, Campbell and Murphy reported that insomnia was present in 29% of menopausal women with HFs vs. 11% in those without HFs [34].

### 4.3. Mood Disorders

The relationship between sleep and mood disorders is well established in the general population. It has been reported that up to 90% of patients with major depressive disorder have sleep problems [35], and that symptoms of depression and anxiety are associated with self-reported poor sleep, in the setting of a non-psychiatric population [36]. Women are at increased risk of developing major depressive disorder during the menopausal transition, especially if they suffer from HFs [10]. It was postulated that HFs disrupt sleep, intrusive anxious thoughts during nocturnal awakenings trigger daytime mood symptoms (domino effect), and depression links with insomnia, in a vicious cycle [37]. Additionally, Vousoura et al. found that HFs and depressive symptoms were associated with different patterns of sleep disturbance, with HFs being related to frequent awakening during sleep, whereas depression was uniquely associated with difficulty falling asleep and waking up earlier than desired [38].

### 4.4. Circadian Rhythm Modifications/Decreased Melatonin Secretion

Circadian rhythm is an internal biologic clock for commencing and monitoring of various physiological processes, including the sleep/wake time schedule. This circadian pacemaker, located in the hypothalamic suprachiasmatic nuclei and the circadian clock, undergoes substantial changes throughout life. Melatonin, synthesized in the pineal gland, also regulates the circadian rhythm by detecting changes in the length of day and night or seasonal sunlight hours [39]. Aging itself is associated with both circadian rhythm alterations and decreased melatonin secretion and, in particular, women have been shown to experience greater sleep difficulties—difficulty falling asleep and waking up earlier than desired—due to a significant decrease in melatonin levels following menopause as well as aging [18,39]. Sex steroids modulate the sleep-favoring effects of melatonin, such as peripheral vasodilation and thermoregulation; therefore, in menopausal women, the decline in female hormones facilitates the occurrence of insomnia [40]. Additionally, the gradual decrease in melatonin along with the steep decrease in estrogen concentration during menopause have been reported to contribute to insomnia in postmenopausal women [41].

## 5. Assessment of Insomnia in Menopausal Women

The diagnosis of insomnia is mainly performed clinically based on the subjective complaints of the patient, and in menopausal women, insomnia commonly occurs as a secondary disorder to physical and psychiatric problems, underlying other sleep disorders, such as OSA or RLS [10,11]. Therefore, careful assessment by proper history taking is important to exclude the comorbid factors. A detailed history from patients and family members using sleep questionnaires and diaries, including the onset of insomnia, pattern and frequency (number of nights/week) of insomnia symptoms, sleep/awake schedule, frequency and bother from menopausal symptoms (HFs and night sweats), and contributing factors or diseases, should be performed. The impact of the sleep complaint on the patient’s life, daytime sleepiness, sleep hygiene, and physical symptoms—snoring, any apneic episodes, dryness of mouth, sweating, restless legs sensation, and periodic limb movements—suggesting other sleep disorders, such as OSA, RLS, etc., should be assessed. The medical, surgical, and psychiatric history, and medications and caffeine/alcohol/nicotine/illicit drug use, are also reviewed [11,42]. Although a polysomnography (PSG) is not generally an essential test for the assessment of insomnia, in menopausal women suffering from persisting sleep disturbances suggesting primary insomnia or other sleep disorders, such as OSA, PSG and a comprehensive assessment are needed [10,43]. Detailed contents of these comorbid diseases with sleep disorders are considered outside the scope of this review, which highlights primary or secondary insomnia in postmenopausal women.

## 6. Management of Insomnia in Menopausal Women

**Figure 1:**
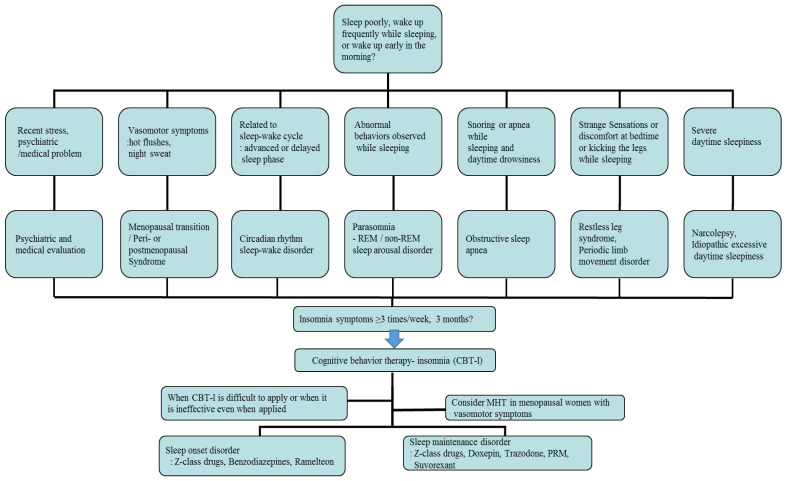
An example of a flowchart for diagnosing and treating insomnia, reflecting patient history and related symptoms, in postmenopausal women. REM: rapid eye movement; MHT: menopausal hormone therapy; PRM: prolonged-release melatonin.

First of all, individual treatment for the identified underlying disease or condition of insomnia is generally necessary, but insomnia after the menopausal transition may be associated with multiple overlapping factors, so management can be complicated and requires individualized treatment. In cases related with multiple sleep disorder factors, such as moderate to severe HFs, depression, and OSA, combined treatments can be considered. The main treatment options for menopausal women with insomnia include non-pharmacological treatment and pharmacological treatment, which are represented by cognitive behavioral therapy for insomnia (CBT-I) and MHT/non-hormonal pharmacological treatment, respectively [42,44]. According to the clinical practice guidelines of the American Academy of Sleep Medicine (AASM) and the European Sleep Research Society (ESRS) for the treatment of insomnia, CBT-I is the first-line intervention for all patients with chronic insomnia, and similar considerations should be given to menopausal women with insomnia. A pharmacological treatment can be offered if CBT-I is not effective or unavailable [44,45]. In patients with OSA, non-pharmacological therapy—continuous positive airway pressure or an oral appliance—should be applied according to the severity of disorder. In addition, in the cases of RLS, triggering factors such as coffee, nicotine, alcohol, antidepressants, and antihypertensive drugs should firstly be avoided, and dopaminergic agonists are the first-line treatment for moderate to severe disease [10]. An example of a flowchart for diagnosing and treating insomnia in postmenopausal women, reflecting medical history and related symptoms, is provided in Figure 1.

### 6.1. Non-Pharmacological Treatment

CBT-I is a treatment that includes cognitive therapy to change patients’ beliefs and attitudes about sleep and behavioral techniques to improve their insomnia, which typically includes sleep hygiene education to provide better sleep conditions, sleep restriction therapy to increase sleep efficiency, stimulus control therapy to adjust the relationship between sleep and sleep stimulation conditions, relaxation training to promote a good sleep, and cognitive therapy to correct dysfunctional thinking about sleep [46,47]. A meta-analysis and systematic review published by Trauer et al. in 2015 suggested that CBT-I intervention was effective in the general population with chronic insomnia [48]. Recent studies of a randomized controlled trial (RCT) with peri- and post-menopausal women with insomnia have reported that CBT-I improved insomnia and reduced HFs’ interference, as well. CBT-I in menopausal women resulted in significant improvements in self-reported insomnia symptoms, sleep latency, sleep quality, and wake time after sleep compared to the menopause education protocol [49]. In a pooled analysis of data from 4 RCTs, which compared the effects of pharmacologic and non-pharmacologic interventions on insomnia and HFs in 546 peri- and post-menopausal women, CBT-I was also found to be more effective for reducing insomnia symptoms in women with HFs compared with other pharmacologic or exercise treatments [50]. In addition to CBT-I as a non-pharmacological treatment method for insomnia, light therapy and exercise therapy are also expected to be effective in treating insomnia. Light therapy is helpful in stabilizing the circadian rhythm, and exercise therapy is helpful in strengthening sleep homeostasis and improving sleep latency and maintenance [51,52]. The ESRS insomnia treatment guidelines currently suggest light therapy and exercise therapy as adjuvant treatments for insomnia [45], and these can also be considered as non-pharmacological treatment methods. A brief description of CBT-I, the first-line intervention for chronic insomnia patients, is provided in the following sections [46,47].

#### 6.1.1. Sleep Hygiene Education

Sleep hygiene education teaches patients about behavioral and environmental factors to improve sleep, as follows: ensuring the sleep environment is quiet and at a temperature suitable for sleeping, establishing a regular bedtime, adequate exercise and exposure to sunlight, avoiding caffeine in the afternoon and excessive fluids, alcohol, and nicotine at bedtime, limiting naps to 30 min, limiting bedtime screen use, etc.

#### 6.1.2. Sleep Restriction Therapy and Stimulus Control Therapy

These behavioral treatments of insomnia are for breaking the maladaptive connection between going to sleep and the hyperarousal state. Sleep restriction therapy considers the patient’s actual sleep time and limits the time spent in bed to increase the sleep desire. Monitoring the sleep and wake times every day and trying to stay in bed only during sleep can improve sleep efficiency. Stimulus control therapy involves breaking the relationship between being in the bedroom and negative aspects of sleep, such as lying in bed only when tired and using the bed only for sleeping.

#### 6.1.3. Relaxation Training

Relaxation training is used to control thought patterns and somatic tension that interferes with sleep. This relaxation-based intervention, alternating contraction of muscles with relaxation, is achieved through progressive muscle relaxation, abdominal breathing, etc.

#### 6.1.4. Cognitive Therapy

Cognitive therapy examines negative beliefs or dysfunctional thoughts about sleep, such as the belief that insomnia will persist, excessive worry or obsession with sleep, trying to lie down and sleep beforehand, etc., and replaces them with rational thoughts or facts by setting a realistic amount or quality of sleep.

### 6.2. Menopausal Hormone Therapy

Based on the identified roles of reproductive hormones in sleep and the theory that vasomotor symptoms (VMS), such as HFs, in menopause cause insomnia, as discussed above, MHT can be an important treatment for insomnia in menopausal women with hot flashes. Indeed, a meta-analysis including 15,468 women from 42 trials published in 2017 showed that MHT improved sleep quality in menopausal women with VMS, along with improvement in concomitant VMS. There was no significant difference when women without VMS were analyzed separately or combined in this study [53]. However, several previous studies exploring the effects of estrogen and progesterone on sleep efficiency have shown mixed results. While some studies suggested that hormone therapy, such as low-dose estrogen with micronized progesterone or drospirenone, 17β-estradiol-progesterone, and low-dose oral estradiol and venlafaxine, reduced insomnia symptoms compared with a placebo [54,55,56,57,58], some contrary results were also reported, which failed to identify any superiority of MHT over the placebo [59,60]. Even studies favoring the effectiveness of MHT mainly showed improvements in subjective sleep quality and tended to show inconsistent results in objective PSG variables [61,62,63]. This lack of consistency is due to the heterogeneity of the trials regarding differences in study populations, age, definitions of menopausal stages, types of menopause, preparations of hormones, and unstandardized sleep scales; therefore, there is a limitation of overall certainty in the evidence of the MHT effects on sleep disturbances [11,53]. Nevertheless, based on the fact that MHT is effective for HFs in peri- and post-menopausal women and helps improve quality of life, there is an emerging view that MHT can be considered as the first-line treatment when insomnia is suspected to be part of VMS, and it is better to first evaluate the response of MHT and then consider other treatments for insomnia in menopausal women with VMS [64]. In the same context, it was reported that the degree of improvement in VMS was an important predictor of insomnia improvement [65]. Considering the bidirectional relationship between insomnia and depression, it is difficult to determine whether insomnia in postmenopausal women is related to the high prevalence of clinical depression or depressed mood during that period or due to menopause itself. The only way to differentiate may be a trial of MHT and consideration of other therapy if insomnia or depression persists after three months of successful MHT [64]. Before using MHT to relieve insomnia in menopausal women, it is important to monitor the side effects of MHT, such as thromboembolic events and breast cancer, and whether the benefits outweigh the risks should be evaluated. Additionally, there are recent studies showing that transdermal treatment was the safest type of hormone therapy in the assessment of risk of venous thromboembolism [66], and micronized progesterone was more effective for improving sleep, as well as reducing HFs [67], which suggests to consider transdermal estradiol and micronized progesterone for the patients at risk of thromboembolism.

### 6.3. Non-Hormonal Pharmacological Treatment

In the general population, including menopausal women, when it is difficult to apply the recommended first-line treatment of CBT-I or when it is ineffective even when applied, the following non-hormonal pharmacological treatments can be considered for treating insomnia.

#### 6.3.1. Benzodiazepines and Z-Class Drugs

Benzodiazepines (triazolam, temazepam, and estazolam) and Z-class drugs (zolpidem, zopiclone, and zaleplon) act as agonists of the benzodiazepine receptor component of the GABA_A_ receptor complex and are commonly used for treating insomnia. Z-class drugs are known to have relatively fewer side effects compared to benzodiazepines, as they are made to mainly bind selectively to type 1 GABA-A receptors and produce only a sleeping effect [68]. Studies have shown that benzodiazepines and Z-class drugs reduce sleep latency, increase total sleep time, reduce awakenings during sleep, and improve sleep quality [44,69]. Considering the pharmacological properties, Z-class drugs with a short half-life (zolpidem IR and eszopiclone) or short-acting benzodiazepines (triazolam) are used for sleep onset disorder, and Z-class drugs such as zolpidem CR and eszopiclone, long-acting benzodiazepines, and antidepressants are appropriately selected and used for sleep maintenance disorder or early awakening [44,70].

In menopausal women with insomnia, several RCTs for Z-class drugs have also reported that zolpidem increased the total sleep time and decreased the wake time after sleep onset and number of awakenings [71], and eszopiclone was effective in the treatment of insomnia, as well as VMS [72]. However, these agents are suggested for short-term use for ≤4 weeks at the lowest dose in adults with insomnia due to their unproven long-term efficacy, the risk of tolerance, and the potential for dependence and abuse, according to the AASM and ESRS practice guidelines [44,45]. Common side effects of benzodiazepines and Z-class drugs include headache, dizziness, and daytime sleepiness and drowsiness, and in particular, the elderly are at increased risk of side effects such as cognitive function impairment, delirium, and falls and fractures due to the muscle relaxation effect, although Z-class drugs have relatively fewer side effects compared to benzodiazepines [73]. It is not clear to what extent these sedative hypnotics impact insomnia in postmenopausal women, but considering that older women are more vulnerable to fractures, more caution is needed when prescribing them to older women [74]. Indeed, meta-analyses showed that the use of benzodiazepines or Z-class drugs was associated with an increased risk of falls and fractures in older adults [75,76].

Zolpidem, a Z-class drug, is currently the most commonly used drug for chronic insomnia, and the AASM also suggests the use of zolpidem in adults with sleep onset and sleep maintenance disorders. Additionally, benzodiazepines are recommended as the main treatment for some sleep disorders such as REM sleep behavior disorder, restless legs syndrome, and periodic limb movement disorder, while in patients with sleep apnea, the use of benzodiazepines and Z-class drugs can worsen the symptoms [77,78,79].

#### 6.3.2. Antidepressants

Given that insomnia is highly implicated in depression, antidepressants can be considered for treating insomnia in menopausal women with comorbid depression. Moreover, antidepressants, such as serotonin and norepinephrine reuptake inhibitors (SSRIs and SNRIs), are valid treatments for VMS in menopausal women with contraindication to MHT [80,81]. Ensrud et al. reported that escitalopram was effective for insomnia in a RCT of 205 peri- and post-menopausal women with HFs [82]. Mirtazapine is also known to be useful in cases of not only insomnia but also depression, which has a sleep-inducing effect and increased slow-wave sleep effect, along with side effects such as appetite, weight gain, and dry mouth [83]. However, there are limited studies on the direct effects of these antidepressants on insomnia; therefore, using antidepressants to treat sleep disruption for women without depression should not be recommended [70]. Nevertheless, some antidepressants that can currently be used for sleep regulation include doxepin and trazodone. Doxepin is the only tricyclic antidepressant approved by the U.S. Food and Drug Administration (FDA) as a treatment for insomnia. When doxepin was administered at 3 mg or 6 mg, the awakening time after hypnosis, total sleep time, and sleep efficiency were significantly improved compared to the placebo control group, and a meta-analysis demonstrated that it was effective in treating sleep maintenance disorders [84]. Trazodone, a blockade of the serotonin 5-HT2A receptor and histamine H1-adrenergic receptors, was also found to be effective for insomnia in a meta-analysis study [85]. It is used as an off-label sleep medicine at low doses of 25–50 mg, and is known to be helpful for sleep maintenance disorders rather than sleep initiation [86]. Compared to benzodiazepines or Z-class drugs, doxepin and trazodone have an advantage in terms of side effects, as these drugs are less prone to abuse or dependence, and the risk of falling is relatively low, so they may be advantageous when prescribed to the elderly [44,86].

#### 6.3.3. Melatonin

Melatonin basically advances the sleep cycle and has a sleep-favoring effect. Supplementation with melatonin has been reported to improve insomnia symptoms and mood disorders in postmenopausal women without serious side effects [87,88]. A prolonged-release melatonin (PRM) agent (2 mg) was approved for patients with primary insomnia over 55 years old for short-term use in some European countries [89]. Unlike existing melatonin preparations, which did not have sufficient effects due to their short half-life of 35 to 50 min, the prolonged-release formulation can improve the sleep structure by maintaining the concentration over 8 to 10 h, similar to the pattern of melatonin secretion in the body [90]. However, clinical trials among the elderly, including menopausal women, had inconsistent results, not only in the quality of sleep, but also in menopausal symptoms [88,90,91,92]. AASM and ESRS practice guidelines did not recommended the use of melatonin preparations for sleep onset insomnia or sleep maintenance insomnia due to insufficient evidence on their effectiveness, in which the guidelines comprehensively reviewed melatonin without clearly distinguishing between prolonged-release agents and short-acting agents and judging their respective effectiveness. Considering that there are not enough studies conducted on PRM yet, additional research on PRM drugs seems necessary. However, PRM does not act on GABA receptors, so it has fewer side effects, such as cognitive decline, falls, rebound insomnia, dependence, tolerance, and withdrawal symptoms, compared to benzodiazepines or Z-class drugs, making it an effective alternative to conventional sleep drugs for the elderly population, including menopausal women [93].

Ramelteon is a melatonin receptor (MT1 and MT2) agonist that was reported to be effective in improving sleep quality and efficiency as a result of a meta-analysis study, and it was approved by the U.S. FDA for the treatment of insomnia. The AASM practice guidelines also recommended that it can be used for sleep onset insomnia [44].

#### 6.3.4. Orexin Antagonist and Gabapentin

Orexin is a neuropeptide that plays an important role in promoting wakefulness and impairing thermoregulation and plasma level of orexin increases after menopause. Suvorexant, an orexin OX1 and OX2 receptor antagonist, was approved by the U.S. FDA as a treatment for insomnia in 2014, and the AASM suggested that suvorexant could be used for sleep maintenance disorder. Rahman et al. found that suvorexant was a well-tolerated and efficacious treatment for middle-aged women with VMS-associated insomnia, and it reduced VMS in a double-blinded RCT [94].

Gabapentin is widely known as one of the non-hormonal treatments for VMS in menopausal women. Yurcheshen et al. demonstrated that gabapentin improved sleep quality in menopausal women with HFs at a dose of 300 mg, three times daily, in a RCT [95], and positive effects of gabapentin on nighttime awakenings and sleep-enhancing actions were also observed in the hypothesized novel sleep disorder, LUNA, associated with low serum estradiol causing nighttime awakening [96]. Studies on orexin antagonists and gabapentin are limited, so their precise roles need to be further established.

## 7. Conclusions

Diagnosis of insomnia in menopausal women is largely performed based on the subjective complaints of patients and may be complexly related to a variety of causes, including changes in female hormones, aging, weight gain, psychosocial problems, and alcohol and drug use. Careful history taking and multidisciplinary physical and psychosocial evaluation are necessary and, in particular, comorbidities related to sleep disorders, such as OSA, must be taken into consideration. Additionally, a unique aspect of insomnia in postmenopausal women is that menopausal symptoms (HFs, mood disorders, musculoskeletal symptoms, and pain) due to hormonal decline can be closely related to sleep disturbances. Therefore, menopausal hormone therapy (MHT) should be considered as the treatment of choice among pharmacological treatments, following cognitive behavioral therapy, which is suggested as the first-line treatment in the general population insomnia treatment guidelines. Additionally, melatonin and 5HT-based drugs, which have fewer side effects, along with MHT should be preferentially recommended in menopausal women. However, there is still a lack of standards on the general approach and treatment for insomnia in menopausal women, and more large-scale prospective studies are needed for further insight on the roles of various treatments for insomnia in menopausal women. Thus, until then, menopausal women with insomnia also need an individualized approach and treatment (MHT, prolonged-release melatonin, 5HT-based drugs, etc.) under the premise that CBT should be used as the first-line treatment, following the treatment guidelines of insomnia for the general population.

## Figures and Tables

**Table 1:** Etiology of sleep disorders in menopause.

Physiologic/Physical
Age Circadian rhythm modifications Decreased melatonin secretion Female sexual hormone changes Decreased estrogen and progesterone, increased FSH Menopausal symptoms Hot flushes, night sweats Others Bladder problems, Ill health, chronic pain—musculoskeletal disorders, osteoarthritis, fibromyalgia, cancer, etc. Poor sleep hygiene/circumstances Medication, coffee, smoking
**Psychiatric/Psycho-social**
Mood disorder—depression Anxiety Illegal drugs, alcohol intake Others—familial/economic/social problem: stress, bereavement, divorce, unemployment, finances, etc.
**Comorbid diseases with sleep disorders**
Obstructive sleep apnea Restless legs syndrome Periodic limb movement syndrome
**Others**
Circadian rhythm sleep–wake disorder Narcolepsy, idiopathic hypersomnia Parasomnias

FSH: follicle-stimulating hormone.
